# Examining Data-Driven Approaches for Clean Air Advocacy in a Changing Climate: a Scoping Review

**DOI:** 10.1007/s40572-026-00563-5

**Published:** 2026-09-12

**Authors:** Gabriel Okello, Frank Gramsen Kizza, Natasha O’Sullivan, Lambed Tatah, Tolu Oni

**Affiliations:** 1 IMS Epidemiology, University of Cambridge, Cambridge, UK; 2 Makerere University Lung Institute, Kampala, Uganda; 3 African Centre for Clean Air, Kampala, Uganda; 4 Department of Public Health and Primary Care, University of Cambridge, Cambridge, UK

**Keywords:** Clean air, Advocacy, Data-driven approaches, Community action

## Abstract

**Purpose of Review:**

Air pollution remains a global public health and climate challenge, causing millions of premature deaths annually and disproportionately affecting marginalised communities. This scoping review examines how data-driven approaches have been used to advocate for clean air in the context of climate change, what methods exist for data mobilisation, enabling conditions, limitations, and whether there were any equity or health considerations.

**Recent Findings:**

Recent advances in low-cost monitoring, satellite data, and digital platforms have transformed the availability and granularity of air-quality data. However, how these diverse data sources are mobilised and used to influence policy, shape public discourse, and support community action remains poorly understood.

**Summary:**

Fifty studies from 41 countries and over 100 cities were included. Five categories of data-driven approaches were identified. These included sensor-based monitoring, modelling, digital platforms and data visualisation, community-centred approaches, and lived-experience assessments. Most studies (86%, *n* = 43) combined multiple data approaches. Data -driven approaches were used to generate and communicate evidence, support policy engagement, identify inequities, foster collaboration and mobilise communities. This review highlights the need to embed community co-production within monitoring efforts, align funding with long-term governance systems, and standardise open data platforms to ensure that clean-air initiatives are equitable, durable, and responsive to climate and health priorities.

**Supplementary Information:**

The online version contains supplementary material available at https://doi.org/10.1007/s40572-026-00563-5.

## Introduction

Air pollution remains one of the most urgent environmental and public health challenges contributing to more than 8 million premature deaths annually worldwide [1], alongside a substantial health burden, reduced productivity, social inequalities, and the climate crisis [2, 3].

Major pollution sources, including transport, industrial processes, agriculture, waste burning, and household energy use also emit greenhouse gases [4, 5]. Addressing air pollution from these sources, therefore, simultaneously supports climate change mitigation [6]. However, addressing air pollution presents substantial complexity because its sources are diverse and frequently intersect across sectors. Additionally, rapid urbanisation and population growth have intensified demand for transport, energy, and waste systems leading to worsening local pollution [7, 8]. However, air pollution burdens are unevenly distributed. Marginalised and low‑income populations disproportionately experience harmful levels of ambient exposures due to their proximity to pollution sources such as living near major roads [9]. Structural inequities, including inadequate housing, limited healthcare access, and reduced socio‑political power, compound these exposures, resulting in significantly elevated unequal health burdens within these communities [10–12].

Efforts to mitigate air pollution globally are constrained by more than limited access to monitoring data. [13, 14]. Weak regulatory and enforcement capacity, shortages of technical expertise and staff, fragmented institutional mandates, insufficient funding, competing development priorities and resistance from politically or economically powerful sectors can all impede action [5, 15–19], even where evidence is available. Within this wider set of governance constraints, inadequate measurement remains an important barrier. Regulatory-grade monitoring stations are expensive to install, operate and maintain, leaving many regions, particularly in low-income countries, without sufficient monitoring networks to identify pollution hotspots and inform targeted interventions [20]. Recent technological advances are however reshaping this landscape. The proliferation of low-cost air quality sensors, open-source software, and cloud-based data platforms have expanded access to more spatially and temporally granular air quality data [21, 22]. These advances present significant opportunities to strengthen air pollution management and accountability. In parallel, investments by philanthropic foundations, governments, and multilateral organisations in data infrastructure, technical training, and capacity building have also helped to expand monitoring coverage and increase the value of these data [17, 23, 24].

Despite these gains, substantial inequities in coverage, data quality and accessibility persist. Communities most burdened by pollution frequently lack adequate monitoring infrastructure, technical capacity, or mechanisms to act on the evidence [25]. Conventional datasets may also fail to capture the lived experiences, creating blind spots in the design, implementation, and evaluation of air quality interventions. These limitations underscore the need for approaches that integrate high-resolution data with local realities and community priorities.

The value of data extends beyond technical assessment. Data must be actionable, accessible, and empowering for communities if clean air initiatives are to be meaningful and sustainable. Because air pollution sources span many sectors, lasting progress requires data-driven advocacy supported by multistakeholder engagement and political will. The Advocacy Coalition Framework (ACF) underscores this dynamic, highlighting how actors from different sectors can form coalitions that share beliefs and coordinate strategies over time to influence policy change [26]. Within this lens, data functions not merely as evidence but as a strategic resource that coalitions use to frame problems, build consensus, contest opposing narratives, and push for policy reforms.

This scoping review examines the global evolving landscape of data-driven approaches used to advocate for clean air as a public health and climate priority. It synthesises the approaches used to mobilise data for advocacy, the enabling conditions and limitations encountered, documented outcomes, and the extent to which health, climate and equity considerations were incorporated. By critically assessing these approaches, the review highlights proven approaches, lessons learned and best practices that can be replicated and scaled whilst considering local contextual factors. The review also highlights opportunities to strengthen data-driven clean-air advocacy and anticipate potential barriers, helping advance scalable, community-centred approaches capable of sustaining a broad and inclusive clean-air and climate-mitigation movement.

The specific research questions we sought to answer were:

- What data driven approaches have been used to advance clean air in communities?
- How were the data mobilised?
- What were the reported challenges and/or facilitators in mobilising these data?
- What were the reported outcomes?
- Was the data health/climate-relevant and/or equity centred?

## Methods

We conducted a scoping review guided by Arksey and O’Malley’s [27] five-stage framework as the basis of the protocol for conducting this review. We used this approach because a scoping review provides an overview of a vast topic [28] and is a suitable approach for exploring a body of literature to identify knowledge gaps [29]. The geographic scope of this scoping review was global, encompassing evidence from diverse countries and cities worldwide.

### Identifying the Scope of the Review

We (TO, GO and LT) convened two in-person consensus meetings to refine the review scope, agree inclusion parameters, and develop the search domains (Supplementary File S1). Following the pilot, two additional reviewers (FGK and NO) were incorporated into the team to manage the substantial screening workload identified. The multidisciplinary team, spanning air quality science, transport modelling, environmental health, global health, and urban epidemiology, worked iteratively to define constructs, generate and test search terms, and harmonise terminology across domains. The senior author (TO) provided strategic oversight on scope framing and methodological decisions.

We adopted Kennedy’s (2024) definition of advocacy as a process through which individuals or organisations support others to understand and exercise their rights, or to help them articulate their views and needs to decision makers [30]. In this study, advocacy refers to coordinated actions by individuals, communities, organisations, and coalitions to influence policies, practices, and social norms related to air pollution and to advance clean air. This includes public awareness campaigns, policy engagement, participatory monitoring, community mobilisation, strategic communication, and coalition building. Our search strategy was guided by five key themes: air quality, data mobilisation, advocacy, climate co-benefits, health-relevant and equity-centred aspects.

### Identifying Relevant Studies: Eligibility Criteria, Information Sources and Searching

We targeted literature published until July 2025. Searches were run in MEDLINE, CINAHL, Global Health, SCOPUS, Web of Science, and Global Health Africa Index Medicus using controlled vocabulary and free-text terms tailored to each database. Following a pilot in May 2025, final searches were executed in September 2025 (search strategies in Supplementary File S1). We complemented database searches with a structured grey-literature strategy search covering major institutional repositories and organisational websites, including: WHO IRIS, World Bank, Health Effects Institute, OECD, UNEP, ICCT, Clean Air Fund, C40 Cities, Local Governments for Sustainability (ICLEI), OpenAQ, ClientEarth, IQAir, WRI, NRDC, SEI, Vital Strategies, Resilient Cities Network, Rockefeller and Fondation Botnar portals. We also employed backward reference checking and forward citation tracking to identify additional eligible materials.

### Article Selection

Citations were imported into Covidence for screening and workflow management. Title/abstract and full-text screening were conducted in pairs by trained researchers (GO, FGK, NO, LT), with senior reviewers (GO or LT) arbitrating discrepancies. GO independently verified all the level-1 and level-2 screening decisions. Reviews were excluded as study types but mined for eligible primary citations.

Inclusion criteria focused on studies/initiatives that reported:

- Data-driven approaches to advance clean air as a climate and health issue
- Data mobilisation approaches. We adopted the concept of data mobilisation from Diack et al. [31]and Schulman et al. [32] and defined it as the development and implementation of data‑curation processes that enhance the openness, accessibility, and re‑use of data.
- Outcomes from strategies addressing clean air.

Exclusion criteria: Studies that did not report any implemented intervention or actionable initiative to advance clean air and non-English publications due to resource constraints for translation.

### Data Extraction

Full-text study characteristics were tabulated, and data extracted into a spreadsheet organised by domain. We customised and piloted a Covidence extraction form (Supplementary File S2). We extracted eligible studies and double-extracted selected fields from a random 20% of the studies. The group met to resolve the discrepancies when this double-extraction process revealed discrepancies exceeding 50%.

### Data Analysis

The team analysed the data using a broad thematic analysis to develop the sub-themes from each theme. The researchers came together to discuss the themes and key findings across all and within each theme. Final broad themes included:

- Identification and frequency analysis of countries and cities
- Data-driven approaches and respective reported outcomes
- How data was used for advocacy.
- Thematic categorisation of studies including climate focused only, health focused only, and climate and health categories.
- Data mobilisation analysis
- Thematic analysis of reported challenges, facilitators, outcomes, health and equity considerations.

## Results

**Fig. 1:**
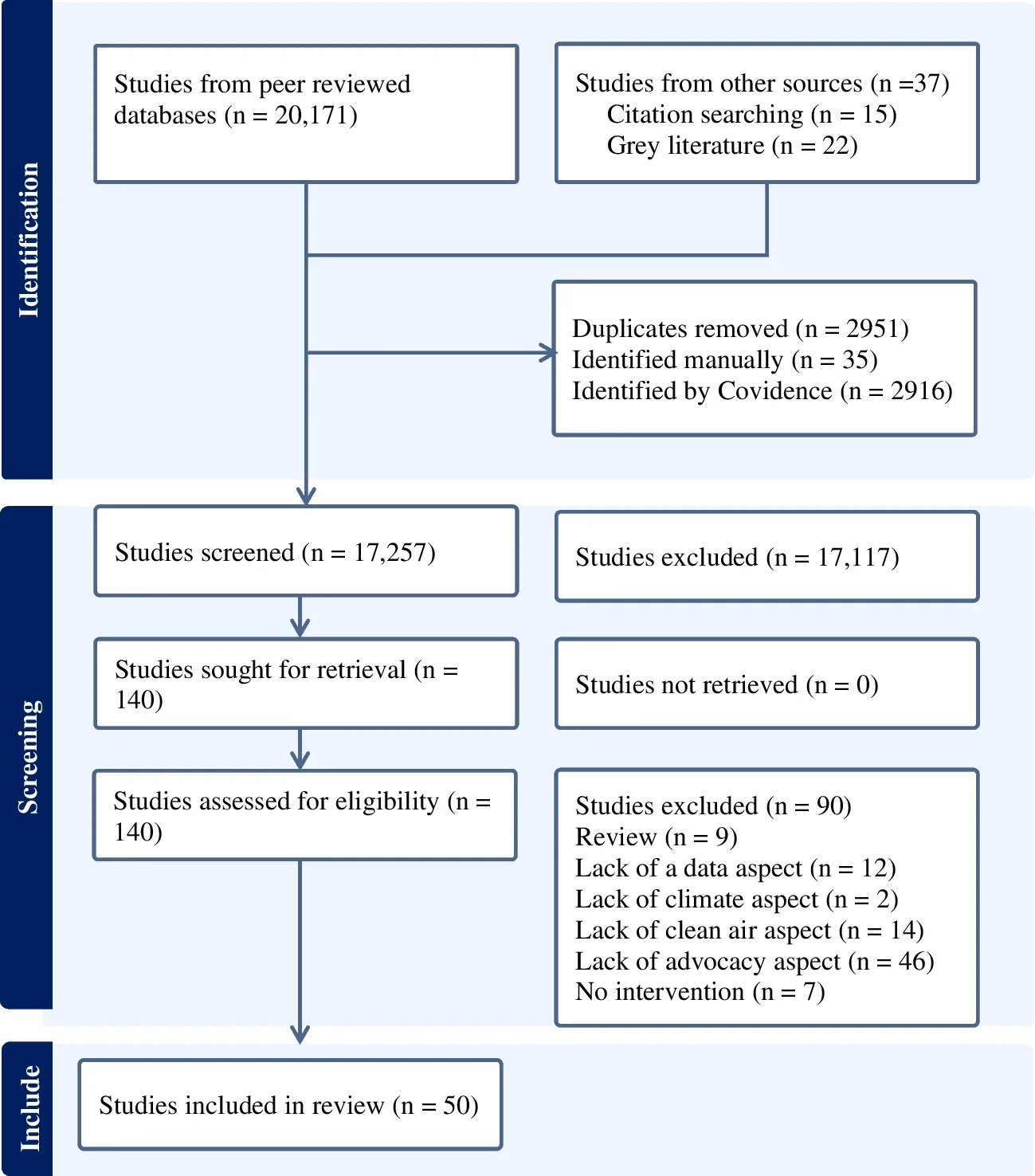
PRISMA Flow Diagram for scoping review showing literature search and selection

Our initial search yielded a total of 20,208 articles and reports. We identified an additional 37 articles and reports through reference chaining and grey literature. From these, 140 studies met the criteria for full-text screening. A final subset of 50 studies and reports met the eligibility criteria and were eventually included in the review (Fig. 1). Of these, 78% (n = 39) were academic manuscripts, whereas 22% (n = 11) were grey literature. Studies represented diverse formats, including peer-reviewed publications, program evaluations, case studies, annual reports, and policy documents. The majority of studies (*n* = 39; 78%) were published in recent years (2020–2025), reflecting growing momentum in data-driven clean air initiatives globally.

### Geographic Distribution

#### Countries

**Fig. 2:**
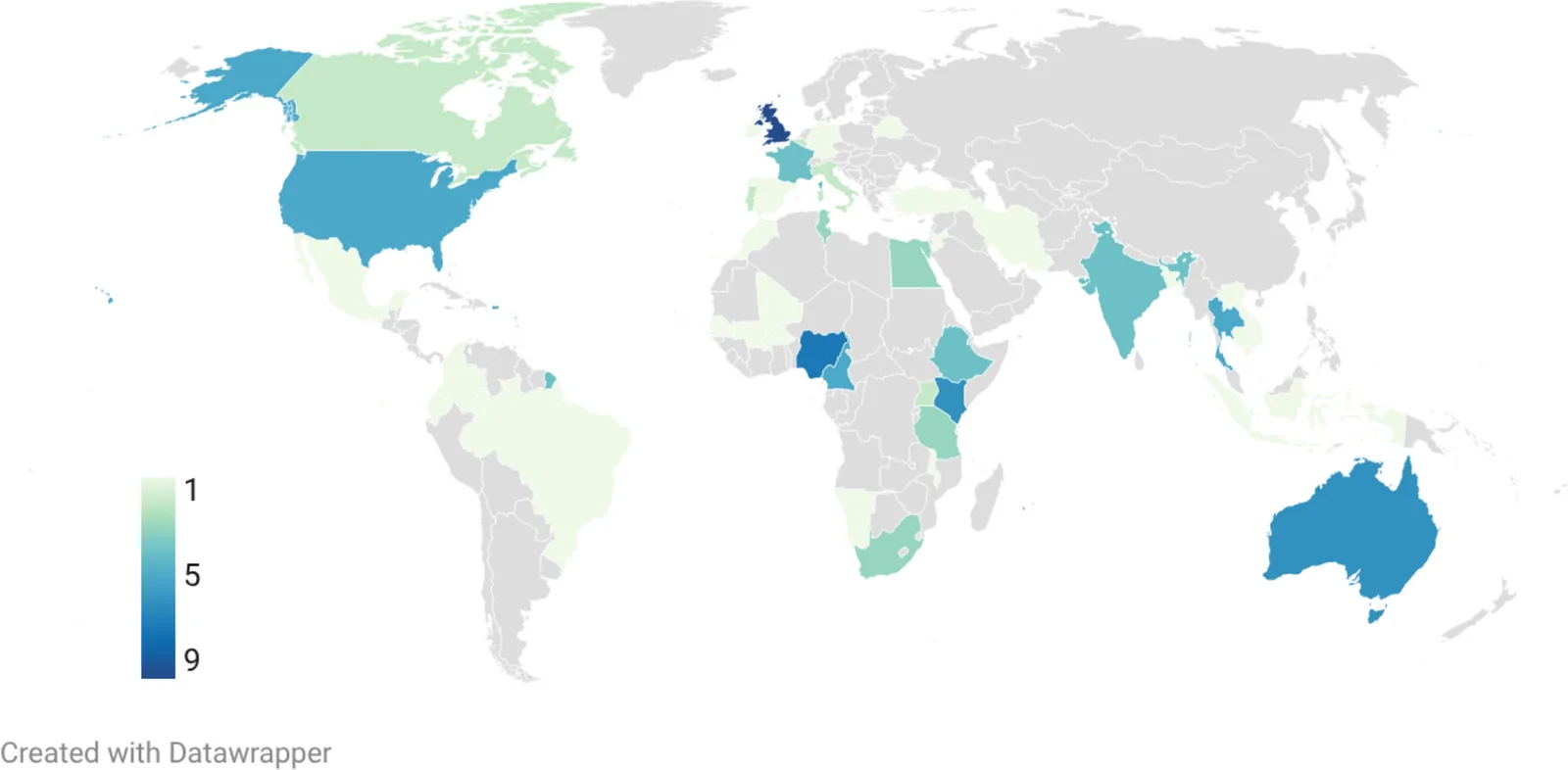
Distribution of data-driven clean air initiatives by country. *(Map created by the authors using Datawrapper)*

Data‑driven clean‑air initiatives were identified in 41 countries across six continents (Table 1). These efforts ranged from multi‑country collaborations to regional and locally embedded programmes, reflecting broad geographic diversity. 41 countries (Fig. 2) were identified as implementation sites for data-driven clean air initiatives.

**Table 1:** Distribution of data-driven initiatives for clean air

Continent/Region	Countries	Studies
Africa	Ghana (*n* = 4), Kenya (*n* = 4), Nigeria (*n* = 4), South Africa (*n* = 4), Uganda (*n* = 3), Cameroon (*n* = 3), Ethiopia (*n* = 2), The Gambia (*n* = 2), Benin (*n* = 1), Burkina Faso (*n* = 1), Côte d’Ivoire (*n* = 1), Madagascar (*n* = 1), Morocco (*n* = 1), Republic of the Congo (*n* = 1), Rwanda (*n* = 1)	Bainomugisha, [33], Clean Air Fund [34], Urban Better, [35], Clean Air Fund, [36], Clean Air Fund, [37], Gwaze, [38], Blaszczyk, [39], Clean Air Fund, [40], Belachew, [41], Bonell, [42], Awokola, [43]
Asia	China (*n* = 7), India (*n* = 5), Indonesia (*n* = 4), South Korea (*n* = 3), Bangladesh (*n* = 1), Japan (*n* = 1), Mongolia (*n* = 1), Nepal (*n* = 1), Taiwan (*n* = 1), Thailand (*n* = 1)	Ng, [44], Wang, [45], Yu, [46], Zhang, [47], Banerjee, [48], Bijal Brahmbhatt, [49], Kumar, [50], Bößner, [51], Clean Air Fund, [40], Blaszczyk, [39], (Park, [52], Relvas, [53], Yorifuji, [54], Clean Air Fund, [34], Clements, [55], Liao, [56], Clean Air Fund, [37]
Europe	United Kingdom (*n* = 7), Poland (*n* = 4), Belgium (*n* = 2), Italy (*n* = 2), Spain (*n* = 2), Bulgaria (*n* = 1), France (*n* = 1), Netherlands (*n* = 1), Portugal (*n* = 1)	Burbi, [57], Rogers, [18], Okeowo, [58], Simpson, [59], Frankowski, [60], Maltby, [19], Blaszczyk [39], Salas, [61], Clean Air Fund, [37], Panteliadis, [62], Relvas, [53]
North America	United States (*n* = 13), Mexico (*n* = 2)	Bland, [63], Brooks, [64], Castillo, [65], Castner, [66], Champion, [67], Clean Air Fund, [40], Daouda, [68], Herbert, [69], Kelly, [70], Kotcher, [71], Lau, [72], Slavik, [73], Symanski, [74], Clean Air Fund, [34], Clean Air Fund, [37]
South America	Colombia (*n* = 4), Brazil (*n* = 2), Chile (*n* = 2)	Clean Air Fund, [34], Clean Air Fund, [37], Clean Air Fund, [40], Gutiérrez, [75], Royo, [76], Relvas, [53], Ruiz-Tagle, [77]
Global/multi-country	Global (*n* = 2), European Union (regional) (*n* = 2)	Boulianne, [78], (WHO, [79], Clean Air Fund, [37, 80]

### Data-Driven Approaches for Clean Air and their Respective Reported Outcomes

The included studies used a range of data-driven approaches to support clean air advocacy and often used them in combination to maximise impact (Table 2). Nearly half of the studies (*n* = 22; 44%) employed a combination of two methodological approaches. A further 22% (*n* = 11) utilised more than three approaches, while 20% (*n* = 10) implemented three distinct approaches. Only a small proportion (*n* = 7; 14%) relied on a single approach.; Approaches adopted included the following.

**Table 2:** Data driven approaches for clean air and their respective reported outcomes

First author (Year)	Country	Data-driven approaches	Reported outcome
Rogers [18]	United Kingdom	Assessment of experiences using interviews with stakeholders -Analysis of opinion polls, media mentions	-The issue gained national prominence, enabling new initiatives and reframing insulation as a social justice concern; however, it also triggered backlash that heightened reputational risks and polarisation -Impact attribution remained difficult amid the energy crisis and increased stakeholder reluctance to engage
Bainomugisha [33]	Uganda, Kenya, Nigeria, Cameroon, the Ivory Coast, Madagscar	-Sensor-based monitoring, including custom-made low-cost sensors to monitor air quality -Digital platform to show air quality data -Community engagement	-Available air quality datasets for national and city policy and regulatory development.—National air-quality standards developed in Uganda and city-specific air quality action plans in Jinja, Nairobi, Kisumu, Kampala.—Mobile App and dashboard to access to real-time air-quality information. -Large-scale public awareness via campaigns, community events, and media broadcasts.—Open datasets to support studies on health impacts, and environmental modelling. -Development of machine learning models for sensor calibration and large-scale monitoring
Awokola [43]	Gambia; Kenya; Uganda; Benin; Burkina Faso; Cameroon Nigeria	Sensor-based monitoring using Local low-cost sensors	Documentation of practical and feasible ways to use low-cost air quality sensors to produce a network of air pollution data across several sub-Saharan African countries
Gwaze [38]	South Africa	Sensor based monitoring and digital platforms	Mobile application tool (South African Air Quality Information System mobile application) providing real time state of air quality to South Africans
Panteliadis [62]	Netherlands	Sensor based monitoring and modelling approaches	Significant reductions in traffic-related pollution after Low Emissions Zones implementation
Brahmbhatt [49]	India	-Community engagement through training women as Air Quality Index (AQI) ambassadors -Sensor based monitoring	- Development of the Green Delhi app to support in alerting authorities to pollution issues for faster resolution. -People lost jobs because of the construction ban in November 2022. Government supported the compensation of people left without jobs during the construction ban in November 2022
Blaszczyk [39]	Indonesia; South Africa; Poland; UK	Community engagement through photojournalism	-Publicly available Climate Visuals photo collection (over 200) showing community experiences of air pollution increasing visibility inequities and integrating evidence-based images into outreach and policy engagement
WHO [79]	Global	Community engagement through data visualization	Raised political commitment, over 70 jurisdictions joined BreatheLife, and hundreds of thousands of unique visitors to digital platforms
UrbanBetter [35]	Nigeria	Sensor based monitoring using mobile sensors during runs	-Raised awareness among community members. -Identified spatial inequality in air quality. -Strengthened community engagement and visibility of pollution data
Clean Air Fund [37]	European Union, Ghana Thailand, Colombia, Indonesia, South Africa, UK, Mexico, Kenya, Paris France, Brazil, Bulgaria, Poland, Italy, Belgium, Poland, India,	-Community engagement -Air quality sensor networks -Surveys	-EU adopted a stronger Ambient Air Quality Directive and resolution on climate and health adopted by WHO -World Bank pledged $1 billion for clean air. -Clean Air Fund influenced $11.8 million funding −100 clean air policies implemented by cities through the Clean Air Accelerator. −14 cities committed to cutting air pollution and climate emissions by 30% by 2030. -Krakow saw over 100 fewer smog days annually. -London's ULEZ reduced NO_X_ emissions by 24% and particulate matter exhaust emissions by 29% -Launch of AQMx, knowledge platform to access guidance and tools on air pollution management -Independent evaluation of 134 air quality sensors in the global south -Landmark resolution mandating Africa’s first Clean Air Action Plan passed by the UN Environment Assembly. -National and local advances included Ghana’s new Environmental Protection Act, a regional airshed approach in India, Poland’s ban on highly polluting coal heaters, expanded low emission zones, Nairobi’s draft air quality regulations, groundwork for a Clean Air Zone in Johannesburg, Accra’s advancing action plan, improved monitoring in Jakarta, and pilot initiatives in Delhi, Agra, and Gorakhpur to curb waste burning, promote green infrastructure, and reduce transport and construction emissions
Clean Air Fund [40]	Ghana; Colombia; China; Indonesia; Uganda; USA	-Sensor-based monitoring methods involving air quality monitoring and environmental health surveillance -Emission inventories, data analysis and modelling approaches -Multisectoral engagement	-Shifts to LPG, biogas, and electricity; cleaner transport; expanded recycling and composting; restoration of green urban spaces that promote active mobility; bans on residential coal; and reduced illegal waste burning. -Pollution reductions in Beijing (PM_2.5_ decreased 66.5%, SO_2_ 88.7%, NO_2_ 58.9%, PM_10_ 50% between 2013 and 2022 and New York (PM_2.5_ 40%, NO_2_ by 38%, NO by 58%, SO_2_ 97% between 2008 and 2021. -Kampala expanded monitoring, raised public awareness and launched car-free days; Ghana added black carbon to its 2020 GHG inventory and trained health advocates. In Barranquilla, Todos al Parque and related initiatives restored extensive green space and wetland access.—Increased multisectoral engagement, public attention to air pollution, and one community shut down an industrial polluter
Clean Air Fund [34]	EU, Chile, Mongolia, Mexico, China, Ghana, India, South Korea, Bangladesh, Nigeria, China; UK, China, and Nigeria	-Modelling approaches through cost benefit analysis - Assessments of experiences through surveys and impact evaluations for initiatives	-Ultra-Low Emission Zone cut NO₂ by 44%, PM_2.5_ by 27%, and CO₂ by 6%, -Significant rise in public satisfaction with environmental action -Chile, Ghana, Mexico, China reported progress in reducing short-lived climate pollutants, strengthening emissions standards, and improving policy coherence. -Increased energy security, prevention of crop loss, increased productivity, and reduced healthcare expenditure
Kumar [50]	India	Sensor-based monitoring methods using Stove Use Monitoring -Community engagement	-High fuel stacking: None of the households used LPG for more than 55% of their cooking time which meant that health benefits from cleaner cooking were not fully realized -LPG refill transportation, perception of faster cooking, and caste were reported as significant predictors of LPG use
Clean Air Fund [36]	South Africa, Kenya, Morocco, Rwanda, Ethiopia, Ghana, Congo Brazaville, Cameroon, Nigeria	-Sensor-based methods through air quality monitoring networks with satellite remote sensing, forecasting and modelling - External technical assistance to close data and capacity gaps	-Car-free days in Kigali cut PM_2.5_ by 15% on designated days, while expanded monitoring in Johannesburg strengthened decision-making and broadened low-cost sensor coverage alongside Kampala. -Addis Ababa’s promotion of electric cooking contributed to 63% of households shifting to electricity for primary cooking, reducing household emissions. -Kumasi’s composting plant processes 1,200 tonnes of waste daily, improving environmental conditions and supporting agricultural reuse -Across cities including Nairobi, progress includes stronger policies, participatory monitoring, greater public awareness and engagement, enhanced technical capacity, and increased funding for pilot projects
Park [52]	Republic of Korea	-Assessment of experiences through surveys, modelling and data analysis	The coupon program had an adverse effect on fuel switching to clean energy
Bößner [81]	Indonesia	Community engagement	Installation of biogas digesters
Frankowski [60]	Poland	Assessment of experiences through surveys and interviews	-Stove replacement improved indoor air quality for 75%, cleanliness for 61%, added free time for 92% of households, and enhanced thermal comfort for 82%
Kotcher [71]	U.S.A	Assessment of lived experiences through survey approaches	Climate and health advocacy messages were most effective when they combined information about risks and solutions with a normative appeal
Ng [44]	China	-Sensor based and modelling approaches -Assessment of lived experiences through interviews	Marine vessels, especially container ships, responsible for 80 to 82% of emissions in 2007, were identified as major sources of SO₂, NOx, and PM_10_. The Fair Winds Charter secured voluntary low-sulfur fuel use at berth, cutting SO₂ and PM_10_ by about 80%, prompting government action on ship and port emissions and raising public awareness through scientific evidence
Relvas [53]	UK, Italy, Belgium, Spain, Portugal, Korea, Brazil	-Sensor-based monitoring and modelling using sensor data with satellite data	Public awareness of air pollution grew as citizen involvement improved data collection, enhanced decision-making through identifying hotspots, and deepened understanding via co-creation, integrated approaches, and digital twins
Daouda [68]	United States	Sensor-based monitoring and community engagement	Improvement in indoor air quality, improvement in quality of life, all households adopted the intervention
Burbi [57]	UK	-Assessment of lived experiences	The Rapid Farm Practices Appraisal (RFPA) tool was created to assess farm practices based on their mitigation potential
Royo [76]	Colombia	Assessment of lived experiences through surveys, interviews	Co-creation of the EcoLogistics tool facilitating the creation of a low-carbon urban freight action plan, emphasising fleet electrification
Champion [67]	USA	Community engagement and assessment of lived experiences	A new framework was proposed for identifying appropriate heating alternatives that balance health/environmental impacts with local culture, perception, and technical assessment
Gutierrez [75]	Colombia	Assessment of experiences through direct observation, interviews, and documentary evidence	The program distributed over 5200 Improved cook stoveS units between 2010 and 2018. An official report claimed that eco-efficient stoves reduce firewood consumption by 60% compared to traditional stoves. Some adopted the stove while others did not use it
Clements [55]	Nepal	Assessment of experiences using survey approaches	-Reduced air pollution and cooking time -High level of fuel stacking observed and limited adoption due to unreliable electricity supply
Herbert [69]	USA	-Assessment of experience based on survey, interviews -Sensor based methods using indoor air quality sensors	-The pilot led to revisions in protocols for app deployment, sensor installation, and air cleaner distribution for the longitudinal study. -The study generated unexpected insights and encouraged rethinking of approaches, improving the ability to reach shared goals through community engagement
Ruiz-Tagle [77]	Chile	-Sensor based monitoring -Assessment of experiences through surveys	-The intervention reduced residential PM_2.5_ by 10.8% in the treatment group, with no significant indoor changes, and proved highly cost-effective at about USD 9.32 per household, far less than the cost of a wood stove
Belachew [41]	Ethiopia	Assessment of experiences through surveys, interviews	Increased employment, reduced household costs, fuelwood use, cooking time, fire risks, improved social networks, boosted crop production, and decreased forest degradation
Simpson [59]	UK	Assessment of experiences through surveys and interviews	Prioritised list of emissions-mitigation actions, highlighting travel policy as short-term and communication, training, outreach, leadership long-term, with remote consultations and a transport coordinator ranked highly for impact
Salas [61]	Spain	Sensor-based monitoring	-Reduction in NO_2_ levels in Madrid Central with positive spillover effects in neighbouring areas -Increased Public Transport Use
Boulianne [78]	Global	Assessment of experiences through social media	No outcomes reported
Liao [56]	Taiwan	Assessment of experiences through questionnaires	Social influence and perceived climate risk were the strongest levers for behaviour change, media pushes alone were insufficient
Kelly [70]	USA	Modelling approaches by using publicly accessible, environmental databases, emissions and health data	Public access to environmental data expanded, supported by CalEnviroScreen, which became a leading Environmental Justice (EJ) mapping tool and model for other states and Justice40. EJ advocates gained policy influence, formal standing in state processes, improved emissions data, and greater visibility for their claims. Court rulings and AB 617 advanced new air-monitoring and protection regulations
Yu [46]	China	Modelling approaches through satellite-based data	-Reduced PM_2.5_ concentrations by 2.7% and PM_10_ concentrations by 2.5% with heavily polluting enterprises were forced to shut down and prompted local authorities to increase attention and environmental spending
Maltby [19]	Poland	-Sensor based monitoring through hyperlocal sensors and apps and assessment of experiences through Surveys, interviews	-Significant increase in national-level citizen awareness of air pollution and its causes. -Successful legislative changes at the local/regional level, including bans on solid fuels and anti-smog resolutions -Launch of the national Clean Air Programme and increased government focus on gradual improvement in air quality
Wang [45]	China	-Modelling approaches for aggregated data -Analysis of social media posts	A 10% rise in environment related social-media posts corresponded with a 2.7% reduction in PM₂.₅ levels, with 25% of this effect mediated through stronger government regulation. Regions with higher social-media engagement, typically wealthier and more educated, experienced greater pollution declines
Bland [63]	USA	Community engagement approaches	Over a dozen pediatric sites replicated the model, with regions tailoring prescriptions to addressing climate-related health risks and advocacy efforts
Brooks [64]	USA	Community engagement approaches	No reported outcomes
Bonell [42]	The Gambia	Community engagement approaches	-Peer-to-peer teaching, learning and promotion of alternative fuel for cooking made from locally available materials
Symanski [74]	USA	-Sensor-based monitoring -Community engagement -Assessment of experiences	Reduced metal emissions as community awareness, knowledge, and communication improved, enabling best practices, stronger policies, and empowered action on environmental health risks
Castillo [65]	USA	Community engagement approaches	Not measured
Zhang [47]	China	-Sensor-based monitoring -Modelling approaches	-Policy reforms in Hong Kong and China cut shipping emissions, lowered SO₂ at terminals, improved air quality, and fostered long-term cleaner shipping through stakeholder collaboration
Slavik [73]	USA	Community engagement	Not measured
Okeowo [58]	UK	-Sensor-based monitoring and community engagement approaches	The Trust’s CAHF score rose to 37.8%, with the strongest progress in air quality, design, leadership, and travel. CO₂e emissions fell 7%, while indoor PM₂.₅ showed clear daily peaks, particularly during evening visiting hours
Castner [66]	USA	Community engagement approaches	Developed a written report-back intervention, reduced air pollution
Yorifuji [54]	Japan	Sensor-based and modelling approaches	Air quality decline in Tokyo
Lau [72]	USA	-Sensor-based monitoring through citywide air monitoring monitors -Community engagement	Across citywide monitoring and cohort studies, air quality improved substantially: PM₂.₅ fell by up to 51%, NO₂ by 48%, and PAHs by 66%. Annual declines persisted across heating and non-heating seasons, demonstrating sustained, long-term reductions in key pollutants following clean-fuel and emissions-control interventions
Banerjee [48]	India	Assessment of experiences	~ 32% reduction with biomass burning with maximum reduction in sulphate aerosols (47%) and sulphur dioxide (55%) compared to conventional cook stoves
CCAC [80]	Global	Community engagement	Engaging and empowering youth in advocacy and decision-making, Young Scientist Programme

#### Sensor-Based Monitoring

Sensor-based monitoring mainly involved deploying low-cost sensors (fixed and mobile) and hyperlocal monitoring networks across Africa, Asia, Europe, and the United States to reveal pollution hotspots, temporal variations, and real-time exposure patterns. Deployments in Uganda, Kenya, Nigeria, Cameroon, and Madagascar generated locally specific air-quality evidence that was used in community engagement and policy discussions. The source studies reported that these datasets contributed to national standards and city-level action plans, although the strength of attribution was not assessed in this review [33]. In Uganda and several Kenyan cities, sensor datasets underpinned the development of national air-quality standards and city-specific action plans [33]. Long-term monitoring in New York City was used to evaluate pollution-control interventions, and the source study reported sustained reductions in PM_2.5_ (37–51%), NO_2_ (31–48%), and PAHs (66%) over two decades [70]. Comparable initiatives in South Africa [38], The Gambia and East Africa [43], Delhi [49], and Poland [19] demonstrated the scalability of low-cost sensing technologies across varied cultural and infrastructural contexts. Multi-site networks in Ghana and Nigeria facilitated pollution-source attribution, identifying transport and industrial emissions as dominant contributors and catalysing regulatory reform [58, 68].

Mobile and wearable sensors broadened participation by integrating monitoring into everyday physical activity[35], for example, in 2024, UrbanBetter mobilised recreational runners in Lagos to capture neighbourhood-level air-quality data, increasing public visibility of pollution and stimulating local dialogue. Personalised feedback on indoor exposures motivated shifts to cleaner cooking technologies and improved ventilation [68, 69].

#### Modelling Approaches

Modelling approaches involved utilising satellite datasets, emission inventories, and environmental data datasets for mainly forecasting, cost–benefit analysis and evaluation of initiatives. Satellite-derived datasets further addressed monitoring gaps, especially in low-resource settings. Modelling was used to translate observational, satellite and emissions data into forecasts, source-attribution estimates, intervention scenarios and policy-relevant summaries. Several studies reported that these outputs were subsequently used in policy evaluation or regulatory discussions. In the Netherlands and Hong Kong, modelling integrated with sensor data was used to evaluate traffic-related pollution and regulatory outcomes [47, 62]. Similarly, modelling in Japan supported the assessment of diesel-control legislation, providing empirical justification for strengthened standards [54]. Satellite-based emissions analyses in China improved the understanding of pollution trends [46] while emission inventories analysis in Hong Kong revealed contributions of container vessels (80–82%) to marine-source emissions, thus influencing the Fair Winds Charter, which secured voluntary low sulphur fuel use that cut SO₂ and PM_10_ by about 80% [44]. Modelling approaches also guided multisector policy dialogue in Ghana, Colombia, and Indonesia [40], triggered public-health advisories in Delhi and increased public transport uptake by 20% [72].

#### Digital Platforms Approaches

Digital platform approaches involved mobile applications and web-based platforms to improve data visualisation and expand access to air-quality information. In South Africa, an integrated digital platform brought together monitoring data, legislation, and regulatory guidance [38]. WHO’s global visualisation tools were used to present health-risk information to public and policy audiences; the associated source reported increased reach and engagement [79]. Mobile applications supported real-time alerts [56] and hyperlocal response during smog events [19]. Social media platforms facilitated global climate strike mobilisation through the rapid circulation of data-driven narratives [78]. In the UK, analyses of opinion polling and media content showed how data shaped discourse around civil-disobedience campaigns focused on air-quality reforms [18].

#### Community Engagement

Community engagement mainly involved participatory approaches that worked directly with the communities. Training women as AQI ambassadors in Delhi fostered co-production of monitoring evidence while influencing national app development and worker compensation policies [49]. Photojournalism approaches paired imagery with monitoring data to elevate lived experiences in Indonesia and Poland [39]. In the United States, community-led data interpretation strengthened advocacy in environmental-justice contexts [63, 64, 74].

#### Lived Experience Assessments

Lived experience was assessed through survey-based and qualitative approaches. These provided insights into behavioural patterns, perceptions, and policy impacts. Studies in Korea, Poland, the United States, Colombia, and Nepal showed how lived-experience data exposed barriers to cleaner-energy transitions and informed more equitable interventions [52, 60, 66, 75, 76]. Behavioural research demonstrated the effectiveness of risk-communication strategies and policy nudges, such as Chile’s 10.8% reduction in residential PM_2.5_ [77].

### How Data was used for Advocacy

Across included studies, air quality data operated not just as evidence but also as advocacy infrastructure; tools practices, and social arrangements that enabled actors to mobilise the public, influence policy and reshape institutions. Five mechanisms through which advocacy occurred were consistently identified: a) translating complex data into accessible information to influence public awareness and instigate behaviour change; b) increase evidence-based policymaking and regulatory reform; c) highlight inequitable burdens of air pollution; d) institutional change and cross-sector collaboration; e) enhance social and political mobilisation (details in Supplementary File S3).

#### Translation of Information to Impact Public Awareness and Behaviour Change, and Community Mobilisation

A core mechanism through which air quality data was widely used to raise public awareness and mobilise communities to demand cleaner air was through translating technical pollution metrics into understandable information. Mobile applications and public-facing dashboards in particular were used to translate complex scientific information into accessible insights. These tools lowered cognitive barriers and created immediacy, enabling lay public to recognise risk and respond. For example, Liao and colleagues [56] used customised air-quality alarms to increase public engagement, while Maltby et al. [19] and Slavik et al. [73] utilised real-time smog alerts and wildfire smoke communication systems to empower residents to take protective action. UrbanBetter [35] used runners to gather pollution measurements and stimulate local dialogue, while Brahmbhatt and colleagues [49] trained women as AQI ambassadors, linking evidence generation with local leadership. Visual storytelling also emerged as an advocacy tool. Blaszczyk [39] used community co-created photography to generate narratives that made air pollution visible.

A key part of this was the personalisation of the data. Real-time alerts and feedback systems transformed scientific data into a lived experience. This immediacy supported behavioural shifts and interventions. For example, Daouda and colleagues [68] used measured indoor air quality to encourage a transition from gas to electric cooking, while Ruiz-Tagle et al. [77] used real-time feedback as a behavioural nudge to support cleaner household practices [34, 39]. Locally generated evidence informed leaders through forecasts and media [33] whereas citizens and institutions integrated data enabled personalised real-time monitoring that informed practice and policy [53]. Instructional videos that combined cooking demonstrations with air quality data encouraged gas-to-electric stove transitions [68]. Data was also used to develop sector-specific tools for agricultural mitigation planning [41, 48, 57, 75].

#### Evidence-Based Policymaking and Regulatory Reform

Another dominant pathway involved leveraging empirical evidence to shape policy. Data generation created evidence that provided credibility, comparability and accountability that policymakers could not easily dismiss, and also allowed clear metrics for alignment with WHO pollution guidelines. Panteliadis and colleagues [62] utilised monitoring, analysis, and evaluation to track Low Emission Zone (LEZ) impacts, a strategy mirrored by Salas and colleagues [61] for Madrid Central. In Hong Kong, inventory analyses prompted voluntary shifts to low-sulfur fuels [44, 50].

Large-scale, multi-city programmes showed how data can drive systemic reforms. Clean Air Fund reports [34, 36, 37, 40] illustrated how open-data platforms, independent sensor evaluations, pollution-source attribution, and health-economic impact assessments were used to shape ambitious regulatory frameworks, align actions with WHO guidelines, and inform national commitments such as Nationally Determined Contribution revisions[74]. Comprehensive nationwide surveys documented facilitators and barriers to clean-fuel adoption [52]. Multi-stakeholder evaluations identified determinants of sustained LPG use and informed enabling policies [50], while mini-grid trials of electric cooking captured user experience and operational compatibility via exit surveys [55].

#### Institutional Change and Cross-Sector Collaboration

Within institutions, such as hospital and agriculture, data acted as a design and decision-support tool. Okeowo and colleagues [58] documented how hospitals used the Clean Air Hospital Framework to advocate for sustainable procurement and operations while Burbi and colleagues [57] utilised data-driven appraisal tools to shape mitigation options for farmers. Climate and Clean Air Coalition [80] created training programmes that equipped young scientists to use evidence in climate decision-making.

#### Social and Political Mobilisation

In community and political contexts, data serviced as a legitimising mechanism that enabled advocates to direct their efforts toward clearly pollution sources, high-risk areas, and regulatory gaps. By revealing where harms were concentrated and who was responsible, data structured mobilisation and strengthened credibility of environmental justice claims. In London, pollution data was used to support civil disobedience campaigns targeting motorway emissions [18] to increase awareness around insulation as a social justice and climate action issue, while [45] citizen-generated air quality data pressured local governments in China.

Kelly [70] showed how emissions and exposure data guided environmental-justice advocacy, by helping map high-risk neighbourhoods and support new legislation. Symanski et al. [74] used research findings to engage industry and support community-led advocacy in metal-exposed neighbourhoods while Bland et al. [63] embedded environmental exposure screening and guidance into paediatric care, producing decision support tools that both protected families and generated evidence for broader reform.

#### Environmental Justice and Direct Action

Air data quality, pollution hotspot and health data played a central role in environmental justice advocacy by providing communities with the evidence needed to demonstrate disproportionate harms and demand enforcement, for example through high-risk area mapping [70]. Tailored data visualisations and US Environmental Protection Agency (EPA) data strengthened community advocacy, supporting polluting factory closures and redirecting collective action toward traffic-related emissions [66]. Citywide air monitoring informed advocacy for cleaner fuels, underpinning low-emission bus and taxi programs and policies phasing out high-polluting heating oils [72].

## Main Thematic Categories

The studies were grouped into three thematic categories according to their primary focus (Table 3). Nearly half were climate-focused (*n* = 24, 48%). These studies addressed topics such as emissions reduction, pollution monitoring, development of low-emission zones, and broader climate mitigation strategies. Another group of studies integrated climate–health perspective (*n* = 22, 44%), linking air quality to health outcomes.

**Table 3:** Thematic categorisation of studies according to primary focus

Category	Studies
Climate focused	Bainomugisha, [33], Gwaze, [38], Panteliadis, [62], Bijal, [49], Blaszczyk, [39]), Kumar, [50], Frankowski, [60], Ng, [44], (Relvas, [53]), Burbi, [57], Royo, [76], Ruiz-Tagle, [77], Belachew, [41], Salas, [61], Boulianne, [78], Liao, [56], Yu, [46], Maltby, [19], Wang, [45], Bonell, [42], Castillo, [65], Zhang, [47], Castner, [66], CCAC, [80], Park, [52]
Health focused	Slavik, [73], Gutierrez, [75], Clements, [55]
Health and Climate focused	Awokola, [43], WHO,[79], Urban Better, [35], Clean Air Fund, [37], Clean Air Fund [40], Clean Air Fund, [34], Clean Air Fund, [36], Brooks, [64], Daouda, [68], Champion, [67], Herbert, [69], Simpson, [59], Kelly, [70], Bland, [63], Brooks, [64], Symanski, [74], Okeowo, [58], Yorifuji, [54], Lau, [72], Rogers, [18], Bößner, [81], Banerjee, [48]

### Challenges and/or Facilitators Identified in Mobilising and/or Measuring these Data

Across the studies reviewed, challenges in data mobilisation were widespread with nearly all studies (*n* = 44, 88%) reporting barriers. **Technical and infrastructure-related** constraints were among the most frequently reported barriers (*n* = 16, 32%). Several studies documented persistent reliability issues with low-cost sensors [43, 53, 58, 69]. Power interruptions, particularly common in sub-Saharan African settings, disrupted continuous monitoring and undermined data completeness [33, 43, 44, 55, 67, 74, 81]. Further challenges included data transmission failures, insufficient data storage and security systems [33, 49, 69], and the ongoing need for calibration and validation against reference-grade monitors [59]. Limited network connectivity also hindered real-time reporting, reducing the utility of sensor-generated data for timely decision support [33, 43, 69].

Many research teams identified a shortage of **technical expertise and skills gaps** in machine learning, data science, and air quality management [33, 34, 36, 37, 40, 53, 63, 76].. This was compounded by extensive training needs for sensor deployment, maintenance, and data interpretation. Weak institutional capacity and underdeveloped regulatory infrastructures posed additional obstacles [37, 43, 45, 57, 61, 70, 79, 81].

Delays or failures in securing **political commitment** impeded initial implementation in several contexts, while the politicisation of air quality interventions, such as low-emission zones, reduced public trust and policy stability [19, 34, 36, 70]. Regulatory complexity and weak enforcement further diminished the effectiveness of interventions, with some communities and industries actively circumventing restrictions [59, 61].

**Financial constraints** limited the scope and sustainability of monitoring programs, with high costs associated with commissioning high-quality imagery, conducting comprehensive assessments, and maintaining monitoring networks [33, 34, 36, 39, 50, 55, 60, 77]. These constraints were often exacerbated by competing development priorities, particularly in low-income settings.

**Data quality concerns** due to incomplete spatial and temporal coverage, insufficient quality-control mechanisms, limited public data access, and inconsistent methodological standards reduced the comparability and policy relevance of monitoring outputs [34–39, 47, 53, 62, 72, 76, 81]. On the social side, public backlash against advocacy campaigns, community resistance rooted in economic hardship, and job losses associated with pollution control measures created substantial barriers to implementation [35, 40, 45, 46, 50, 56]. Persistent behavioural challenges, such as ineffective communication strategies, including the lack of accessible, relatable imagery, further hindered community engagement [39, 56, 57, 69].

### Facilitators Reported in Mobilising and/or Measuring these Data

Included studies (*n* = 37, 74%) reported several facilitators that enabled successful implementation the reported initiatives. These included:

**Community engagement and capacity building** through training on sensor operation, data interpretation, and advocacy to build local skills and empower residents to participate meaningfully in air quality initiatives. Several studies demonstrated the importance of community champions [49, 66, 74]. Public education campaigns and participatory approaches fostered broader social ownership and strengthened the legitimacy of interventions [47, 53].

**Strong partnerships and collaborative structures:** Many studies benefited from international technical and financial support, particularly from organisations such as the Clean Air Fund, WHO, and the World Bank [34, 37, 40, 49, 58, 79]. Cross-sector collaboration across health, environment, transport, and urban planning sectors were identified as particularly valuable, as was the exchange of knowledge and best practices across cities and countries, which enabled rapid learning and adaptation [40, 58, 59, 79].

**Accessibility of data systems** was a key enabler, with low-cost sensors reducing financial barriers to air quality monitoring and allowing communities to generate higher spatial resolution, locally relevant data that would not have been feasible using conventional regularly systems alone [35, 43]. Digital platforms, such as mobile applications and online dashboards [38, 49, 79], enhanced public access to real-time air quality data [33, 76], supporting both awareness and policy advocacy. Advances in artificial intelligence and machine learning improved sensor calibration while open-data ecosystems [46, 53], contributed to greater transparency and wider use of evidence across researchers, civil society organisations and decision-makers.

**Effective political leadership and policy champions** [59, 63] helped to advance air quality agendas within government, while engagement of health professionals added credibility and urgency to public communication. Legal frameworks mandating monitoring, such as New York City’s Local Law [68, 72], provided clear institutional responsibilities and sustained momentum. Participation in international initiatives, including BreatheLife and C40 Cities networks, offered additional platforms for support [37, 40, 79], visibility, and alignment with global standards.

Finally, **integrated approaches** that foregrounded co-benefits, linking air quality improvements with climate mitigation, economic gains, health benefits, and urban equity, strengthened the case for intervention [19, 34, 39, 40, 53, 55, 56, 58, 60, 61, 72, 79, 81].

#### Reported Health-Relevant and/or Equity-Centred Data

The majority of studies (*n* = 42, 84%) explicitly incorporated health or equity dimensions, underscoring growing recognition of air pollution as both a public health crisis and a social justice issue. Health-relevant approaches often framed air pollution in terms of its global health burden, premature deaths and disease impacts, particularly respiratory and cardiovascular outcomes in highly exposed groups.

Equity-centred approaches were integrated through targeted attention to populations disproportionately affected by air pollution, including low-income households, women, children, the elderly, and marginalised or minority communities [18, 34, 36, 37, 39, 40, 48, 49, 55, 60, 68, 75]. Interventions addressed these inequities through targeted monitoring in disadvantaged areas, community-driven data collection, women-led ambassador programmes [49], expanded green space access[35], and clean cooking initiatives [40, 79, 81]. Several studies emphasised the importance of equity in data governance, including capacity-building efforts in low- and middle-income countries, participatory decision-making processes, and equity-focused funding requirements [37, 43, 79]. Economic equity was also a recurring theme, with studies noting the need to mitigate job losses from pollution control measures, provide compensation where necessary, and ensure that clean energy and mobility options remain affordable.

## Discussion

This scoping review synthesises evidence from 50 studies across more than 41 countries, demonstrating that data‑driven approaches have become fundamental to clean‑air advocacy. The dominance of mixed‑method initiatives reflects the inherent complexity of air pollution, a multisectoral problem shaped by diverse emission sources, local socioeconomic conditions, and political structures. Our findings show that combining quantitative measurements with modelling, digital platforms, and community‑centred approaches enables a more holistic understanding and more effective mobilisation than any single method alone. This aligns with emerging literature emphasising the value of integrated evidence systems for environmental governance and climate‑health decision‑making.

Sensor‑based monitoring was the most widely applied approach and a critical enabler of hyperlocal evidence generation, particularly in low‑resource settings where regulatory networks remain sparse [33, 43, 68]. By revealing fine‑scale temporal and spatial variability, low‑cost sensors expanded the visibility of pollution exposure and created a new entry point for public engagement [33, 35, 69]. This mirrors trends reported in recent global assessments, highlighting the democratisation of data through affordable sensing technologies and open‑source calibration tools [82, 83]. When integrated into broader coalitions, such as policymakers, health systems, and civil society partners, sensor networks informed the design of low‑emission zones [34, 37, 62], revisions to fuel standards [40, 44], and city‑level clean‑air action plans [33, 37, 59]. These findings are consistent with studies demonstrating that hyperlocal data can accelerate regulatory responsiveness and strengthen accountability, especially where communities have historically had limited access to environmental information [84, 85].

Modelling approaches complemented observational datasets by filling spatial and temporal gaps, improving attribution of pollution sources, and generating policy-relevant scenarios [36, 46, 53]. In several contexts, models provided the analytical underpinning for cleaner transport strategies [36], evaluation of industrial emissions [53], or design of household energy transitions [34].

Digital platforms amplified the reach and influence of air‑quality evidence, improving transparency, accelerating information flow, and broadening public access. Dashboards, mobile applications, and global visualisation tools translated complex datasets into accessible formats, supporting real‑time risk communication and enabling individuals and institutions to make informed decisions [33, 35, 38]. The relevance of these tools is increasing as cities adopt open‑data policies and as climate‑related events heighten public interest in exposure information [83, 86]. Social media further extended this reach by facilitating the rapid dissemination of data‑driven narratives, shaping public discourse on environmental justice, climate co‑benefits, and government accountability [45, 78]. These trends align with research showing how digital ecosystems have reshaped advocacy landscapes, making environmental information more participatory, dynamic, and politically salient [87, 88].

Community‑centred approaches strengthened the social legitimacy of air‑quality evidence by integrating lived experiences with quantitative measurements [89, 90]. Participatory monitoring, community data‑interpretation, and visual storytelling methods elevated underrepresented voices and revealed inequitable pollution burdens, particularly in environmental‑justice settings characterised by historical underinvestment and socio‑political marginalisation. These approaches resonate with literature emphasising that evidence‑driven environmental action must be locally grounded, equity‑centred, and co‑produced to be durable [87, 91]. Interventions pairing data with local leadership, including women‑led AQI ambassador programmes and youth training initiatives, exemplify how co‑production can build agency, strengthen trust, and cultivate long‑term advocacy capacity.

Despite these strengths, significant challenges were reported. Technical constraints, including sensor calibration issues, intermittent power, hardware failures, and data quality variability, were widespread, particularly in low‑income and grid‑unstable settings [33, 34, 38, 43, 55]. Capacity gaps in data management, interpretation, modelling and cross-sector coordination also limited the translation of evidence into policy. These challenges mirror longstanding concerns in the literature that low‑cost technologies require sustained investment in calibration, maintenance, and institutional support to achieve policy relevance. Financial constraints further restricted the scalability and long‑term sustainability of monitoring networks, particularly in regions with competing development priorities.

Political barriers, including weak enforcement, regulatory fragmentation, and resistance to interventions perceived as economically disruptive, also constrained progress [18, 19]. Existing research similarly shows that air-quality interventions often confront entrenched political and economic interests, requiring broad coalitions, strategic communication, and an explicit equity framing to achieve impact [92, 93]. Vera [91] documented how "civically valid" monitoring data and an ecosystem of tools underpinned community-based participatory research partnerships between residents, activists, and scientists to hold industry and the state accountable; Richey et al. [92] illustrated how pairing low-cost Wi-Fi sensors with public health data supported collective advocacy in historically under-monitored spaces; and Alegria et al. [93] showed how the Community Resiliency Information Systems (CRIS) platform enabled two-way communication between residents and policymakers within a smart-city framework. High‑income countries benefited from denser monitoring infrastructure, advanced analytic capacity, and stronger regulatory regimes, whereas low‑income countries relied more heavily on participatory methods and low‑cost technologies. This reflects broader geographic inequities noted in global environmental‑health research, where exposure burdens are highest in contexts with the fewest resources for monitoring and governance [93–95].

Equity considerations featured prominently across studies, yet operationalisation remained uneven. Structural inequities, such as energy affordability, job insecurity, and limited political representation, continued to shape both exposure and the uptake of clean‑air interventions. Evidence shows that equity‑centred data mobilisation, when linked with social protection and community decision‑making, enhances both legitimacy and impact. Effective examples included targeted monitoring in high‑exposure neighbourhoods, gender‑responsive capacity‑building programmes, and community‑led advocacy informed by health and exposure data.

## Conclusion

This review highlights the growing importance of data‑driven approaches in clean‑air advocacy and underscores the need to strengthen how evidence is produced and mobilised. The review shows that data-driven approaches can provide infrastructure for advocacy by generating evidence, translating technical information, supporting community participation and facilitating engagement with decision-makers. However, the extent to which these activities directly caused policy or environmental change could not be determined from the included evidence. To maximise impact, three priorities emerge. First, embedding community co‑production in monitoring is essential to ensure data reflects lived realities, builds trust, and empowers affected communities to shape clean‑air action. Second, aligning funding with long‑term governance structures is critical for sustaining monitoring networks, developing institutional capacity, and moving beyond short‑term pilots. Third, standardising open, interoperable data‑sharing platforms will improve transparency, comparability, and cross‑sector coordination. Collectively, these shifts can transform data from isolated measurements into a foundation for scalable, community‑responsive advocacy that drives durable improvements in air quality, equity, and health.

## Key References

- E. Bainomugisha, P. Warigo, F. Daka, A. N.-S. Impacts, and undefined 2024, “AI-driven environmental sensor networks and digital platforms for urban air pollution monitoring and modelling,” *Elsevier*, 2024, Accessed: Mar. 01, 2026. [Online]. Available: https://www.sciencedirect.com/science/article/pii/S2949697724000092.
  - ○ The paper demonstrates how AI-driven low-cost sensor networks, digital air-quality platforms, and community engagement across multiple African countries have generated open, policy-relevant air-quality datasets that directly informed national standards, city action plans, public awareness campaigns, and advanced analytics such as machine-learning calibration models.
- KA Kelly, “The Value of Data in the Fight for Environmental Justice in California,” *journals.sagepub.com Environmental Justice, 2025•journals.sagepub.com*, vol. 18, no. 1, pp. 29–35, Feb. 2025, https://doi.org/10.1089/env.2023.0023.
  - ○ This paper shows how publicly accessible environmental, emissions, and health data can empower environmental-justice advocates, strengthen regulatory action, and expand community influence in shaping air-quality policy and protections.
- Clean Air Fund and Vital Strategies, “Real-world solutions for clean air and health: Six city examples to inspire action on urban air pollution,” 2025. Accessed: Dec. 11, 2025. [Online]. Available: https://static1.squarespace.com/static/651ac816acd03355c91d9e93/t/6638857a9e768817c5eaa531/1714980238179/CaseStudyBooklet.pdf.
  - ○ The report demonstrates how health-economic impact assessments and pollution source attribution are used to align local actions with WHO air quality guidelines.
- Clean Air Fund and Dalberg Advisors, “JOINED-UP ACTION ON AIR POLLUTION & CLIMATE CHANGE Cheaper, faster and fairer,” 2021. Accessed: Dec. 11, 2025. [Online]. Available: https://s40026.pcdn.co/wp-content/uploads/Joined-up_Action_on_Air_Pollution_and_Climate_Change-compressed-2.pdf.
  - ○ This report provides a framework for integrated action. It shows how co-beneficial strategies that address both air quality and climate change can lead to faster and fairer policy outcomes in cities worldwide
- UrbanBetter, “Breathing Battles: Chronicles of Air Quality Advocacy in Lagos.” Accessed: Dec. 11, 2025. [Online]. Available: https://urbanbetter.science/blog/2024/11/08/breathing-battles-chronicles-of-air-quality-advocacy-in-lagos/.
  - ○ This initiatives shows how mobile sensor monitoring during community runs can raise public awareness, reveal spatial inequalities in air quality, and strengthen community engagement by making pollution data visible and locally meaningful.
- M. Daouda, A. Carforo, H. Miller, and J Ventrella, “Out of gas, in with justice: findings from a gas-to-induction pilot in low-income housing in NYC,” *Elsevier Energy Research & Social Science, 2024•Elsevier*, 2024, Accessed: Mar. 01, 2026. [Online]. Available: https://www.sciencedirect.com/science/article/pii/S2214629624002536.
  - ○ This paper shows that clean energy transitions in low‑income housing deliver transformative co‑benefits such as enhancing residents’ quality of life whilst improving indoor air quality.

## Supplementary Information

Below is the link to the electronic supplementary material.

Supplementary file1 (DOCX 16 KB)

Supplementary file2 (DOCX 16 KB)

Supplementary file3 (DOCX 46 KB)

## Data Availability

Data will be made available on request.
