# Supplementary material for: Examining Data-Driven Approaches for Clean Air Advocacy in a Changing Climate: a Scoping Review
Source: Curr Environ Health Rep. 2026 Sep 12;13(1):41. doi: 10.1007/s40572-026-00563-5 (PMC13570917; doi:10.1007/s40572-026-00563-5)
Supplement: Supplementary file 1 [file 40572_2026_563_MOESM1_ESM.docx]

Search domains

Air Pollution:

Clean air/air pollution/air quality: ("air pollution" OR "clean air" OR "air quality" OR "air quality monitoring" OR "climate change" OR "ambient air pollution" OR "indoor air pollution” OR "particulate matter" OR "PM2.5" OR "NO2" OR "Ozone" OR “black carbon” OR “Volatile Organic Compounds”

Advocacy:

"data-driven advocacy" OR "evidence-based advocacy" OR "data mobilisation" OR "data for policy change" OR "data storytelling" OR "data activism" OR "citizen science" OR "community-based monitoring" OR “measure” OR "monitoring” OR “assessment” OR “quantify” OR “assessing” OR “evaluating” OR “mobilise” OR “organise” OR “gather” OR “activate”

Data and mobilisation: "data driven advocacy" OR "advocacy” OR “evidence-based advocacy" OR "data mobilisation" OR "data for policy change" OR "data storytelling" OR "data activism" OR "citizen science" OR "community-based monitoring" OR Measure” OR "monitor” OR “assess” OR “quantify” OR “evaluate” OR “mobilise” OR “organise” OR “Gather” OR “activate” OR political engagement: "public engagement" OR "policy advocacy" OR "political will" OR "public demand" OR "framing"

Climate co-benefits

“climate co-benefits” OR “co benefits” OR “cobenefits” OR “health co-benefits” OR “environmental co-benefits” OR “climate mitigation co-benefits” OR “air quality climate co-benefits” OR “adaptation-mitigation co-benefits” OR “short-lived climate pollutants co-benefits” OR “air pollution reduction health benefits” OR “clean air AND climate benefits” OR “PM2.5 reduction AND climate” OR “health impacts AND climate mitigation” OR “climate policy AND co-benefits” OR “NDCs AND co-benefits” OR “multi-sector climate benefits” OR “integrated climate strategies co-benefits” OR “multiple benefits” OR “synergies climate mitigation” OR “health–climate synergy” OR “co-impact” OR “co-impacts”

Health relevant and equity-centred data: "health equity" OR "environmental justice" OR "health disparities" AND OR "vulnerable populations" OR "equity-centred data" OR "disaggregated data"
