# Supplementary material for: Examining Data-Driven Approaches for Clean Air Advocacy in a Changing Climate: a Scoping Review
Source: Curr Environ Health Rep. 2026 Sep 12;13(1):41. doi: 10.1007/s40572-026-00563-5 (PMC13570917; doi:10.1007/s40572-026-00563-5)
Supplement: Supplementary file 2 [file 40572_2026_563_MOESM2_ESM.docx]

Extraction template

- General information
- Study ID
- Title
- First name of lead author
- Lead author contact details
- Year of publication
- Study Design
- Aim of study
- Type of study (RCT, Cohort study, Cross sectional study, Case report, Evaluation study etc)
- Study population
- Description of population (e.g., specific age groups, school children, youth etc)
- Population size
- Location
- Country or region where the initiative was conducted
- City or cities where the study were conducted
- Inventions/initiatives implemented
- Which Intervention or initiatives were implemented
- Data mobilisation
- How was the data mobilised or measured
- Data driven approaches for advancing clean air
- What specific approaches were used
- What challenges were reported
- What facilitators were reported
- Who was involved (stakeholders involved)
- How were they involved (stakeholders involved)
- Outcomes achieved
- Any reported outcomes
- How were the outcomes measured (answer not measured if there were no measured outcomes)
- Name any health relevant or equity centred aspects considered and/or reported (person extracting should do this)
