# Supplementary material for: Examining Data-Driven Approaches for Clean Air Advocacy in a Changing Climate: a Scoping Review
Source: Curr Environ Health Rep. 2026 Sep 12;13(1):41. doi: 10.1007/s40572-026-00563-5 (PMC13570917; doi:10.1007/s40572-026-00563-5)
Supplement: Supplementary file 3 [file 40572_2026_563_MOESM3_ESM.docx]

| **First author (Year)** | **How data was used for clean air advocacy** |
| --- | --- |
| Rogersetal (2024) | - Data was used for Civil disobedience involving blockading motorways, notably the M25 which encircles London and increase awareness through interviews |
| Bainomugisha (2024) | - Data was used to share evidence of local air quality to leaders and communities through mobile App, forecasts and media broadcasts, and provide training for the stakeholders to understand the evidence and translating complex data into actionable recommendations for policy makers, and communities. |
| Awokola (2020) | - Data was used to increase awareness among with clinical and basic science researchers to measure air quality across 13 locations in Africa |
| Gwaze (2018) | - Data was used to provide one-stop shop tools for all information, from monitoring to legislation, notices, guidelines and contact for air quality officials in different areas across the country. |
| Panteliadis (2014) | - Data was used to monitor, analyse and evaluate the impact of the Low Emission Zone (LEZ) by policy makers, local authorities and researchers. |
| Brahmbhatt (2023) | - Data was used to train women in the community as Air Quality Index (AQI) ambassadors and Involving women ambassadors in collecting data and utilising their voices to create awareness and develop contextually relevant solutions. |
| Blaszczyk (2023) | - Photographers captured images and worked directly with local communities to co-develop stories. |
| WHO (2025) | - Data was used to build High-level coalition building, sharing best practices through online spaces, and implementing public events/webinars |
| UrbanBetter (2024) | - Runners conducted air monitoring, community observations and conversations during runs to promote awareness of air pollution, health impacts and understand local perspectives and barriers. |
| Clean Air Fund (2024) | - Used media (data) to elevate air pollution in Ghana - Utilising air quality monitoring network to identify pollution hotspots and provide smog alerts. - Independent evaluation of 134 air quality sensors from 28 manufacturers - Used surveys to gather feedback from partners - Used data and research to push for ambitious air pollution reduction targets and regulatory frameworks, such as the EU's Ambient Air Quality Directive - Aligning grants with the three drivers of the theory of change: data, demand, and drive to action, ensuring they are evidence-informed |
| Clean Air Fund (2025) | - Communication campaigns and public disclosure of quality information and health advice. Training healthcare workers, media, solid waste sector on effects of air quality - Integrating health goals based on WHO guidelines into air quality management plan - Creating air quality and health working groups to collect evidence on health impacts of air pollution from different sources. - Using local health and economic data to quantify and assess impacts, and cost-benefit analysis to prioritise actions - Using car-free days to promote active mobility and health campaigns. - Leveraging the health sector's network for risk communication -Identifying local leading pollution sources, - Legislation, financial incentives and public education for behavioral changes. - Mapping policies and stakeholders, to enable capacity building, and policy tracking |
| Clean Air Fund (2021) | - Developing frameworks for cities to integrate air quality management and climate action planning. - Implementing national strategies and country action plans. - Revising NDC targets to reduce black carbon by 25% by 2030. - Explicitly integrating air quality goals and cross sector coordination. - Committing funds to support approaches |
| Kumar 2022 (2022) | - Multistakeholder evaluation of factors influencing sustained use of cleaner cooking fuels (LPG) to mitigate household air pollution - Government facilitated participants access to LPG and implemented policies to encourage LPG adoption. |
| Clean Air Fund 2022 (2023) | - Open data platforms to increase access and public engagement e.g mobile applications and data platforms - Governments conducted pollution-attribution and emission-accounting exercises to quantify contributions from various sources. - Awareness and education campaigns e.g car-free days, clean cooking promotion (electric and cookstoves) - Introduction of the Clean Air Bills in 2021 in Nairobi, requiring industries to reduce emissions and aligning with its Climate Action Plan |
| Park 2011 (2011) | - Consumer subsidy program providing briquette coupons to low-income families to alleviate financial burdens - Comprehensive nationwide survey on briquette consumption, purchase methods, price, coupon subsidy scale, reasons for and willingness to switch fuels data collected - Mapping briquette dependence across households to improve policy targeting. - Identifying gaps that block clean-fuel adoption and using socioeconomic profiling to support equitable clean-air policy design. |
| Bößner (2019) | - Stakeholder consultations and engagements through workshop, semi-structured interviews and focus group discussion. |
| Frankowski 2021 (2020) | - Implementing solid fuel stove replacement programs, which assisted households in transitioning towards cleaner energy - Design and implementation of subsidy programs and implementation of anti-smog legislation |
| Kotcher (2021) | - Health information alongside climate and air pollution data presented to allow people provide feedback on the type of messages would motivate them to act on climate change-related issues |
| Ng 2013 (2013) | - Researchers compiled emission inventory analysis and scientific evidence that prompted voluntary industry fuel‑switching to low‑sulphur (≤0.5%) fuels. - Policy makers engaged in policy discussions and proposed regulations |
| Relvas 2025 (2025) | - Citizens and institutions collected and integrated air‑quality data using sensors and AI data‑fusion methods, enabling personalised and real-time urban monitoring and improving sensor performance to inform practice, support policy and management. |
| Daouda (2024) | - Instructional videos by a local chef in both English and Spanish, measured air quality data were used to encourage participants to switch from gas-to-electric stoves |
| Burbi (2019) | - Rapid Farm Practices Appraisal (RFPA) tool was developed to enable science-based assessment of farm practices against greenhouse-gas mitigation strategies. - Insights on challenges, barriers, and motivations related to adopting low-emission practices shaped the practical mitigation options were realistic and farmer-driven. |
| Royo (2025) | - Data was used to track urban freight emissions and evaluate decarbonisation strategies e.g fleet electrification and modifying the electricity grid's carbon intensity |
| Champion 2017 (2017) | - Data was used to identify heating and rate alternatives, cultural assessment, community perception, planning, action, and reflection - Modelling tools were used to determine technical assessments, discrepancies and areas of alignment |
| Gutierrez (2023) | - Data was used to formulate, implement, promote and evaluate transition to improved cookstoves program - Data also helped gather insights into the design practices program's and adoption rates |
| Clements (2020) | - Participants were given electric cookers and induction compatible cookware to test the usage of electric cooking using mini grids. Exit surveys provided feedback on cooking experiences and compatibility of the mini grids for cooking |
| Herbert (2023) | - Sensor used to identify microenvironments with higher exposure with a smartphone app providing current and projected Air Quality Index and educational modules for improving indoor air quality - Community program, designed the pilot study which identified local needs and priorities - Data was also used to train community Ambassadors who provided support for participants. |
| Ruiz-Tagle (2021) | - Data was used as a behavioural nudge with information flyer as a reminder. The flyer explained the information signs. - Data was used to implement policies to lower ambient air pollution - Real-time feedback supported action. |
| Belachew (2024) | - Government led dissemination of improved cookstoves scheme to households. |
| Simpson (2022) | - Data was used to identify, appraise and prioritise potential air quality actions, policy documentation and action trackers. |
| Salas 2021 (2021) | - Data was used in Implementation of Madrid Central, a low-emission zone (LEZ) \| |
| Boulianne 2020 (2020) | - Data enabled sharing messages and images, using hashtags to build shared attention on climate change, coordinate global School Strike 4 Climate protests, and document widespread public discontent. |
| Liao (2019) | - Data allowed users to set customised alarms for local air quality and notified users when hourly monitoring values reach set thresholds and allow to inquire emission data and receive alerts Participants completed the survey on determinants of willingness to act on air-pollution control |
| Kelly (2025) | - Data on emissions, health, and pollution hotspots informed environmental justice advocacy by validating community concerns, guiding mapping of high‑risk areas, shaping climate and air‑quality legislation, supporting cap-and-trade oversight, and strengthening regulatory actions. |
| Yu 2024 (2024) | - Data was used to highlight pollution trends, strengthen accountability mechanisms, and inform evidence‑based regulatory and policy interventions. |
| Maltby (2024) | - Data was used for activist Campaigns, Smog Alert engaged in public awareness campaigns, protests, lobby for legislative change, media campaigns, and collaborations with public sector law groups - Real-time monitoring and Apps facilitated real-time information alerts to the public. |
| Wang (2021) | - Data enabled citizens to pressure local governments, whose regulatory responses, monitored by central authorities, partially mediated pollution reductions, demonstrating how social‑media data and city‑level indicators supported advocacy for improved air‑quality governance. |
| Bland 2025 (2025) | - Data was used to inform a clinical tool integrating environmental health into pediatric care, enabling screening for needs, delivering guidance, and connecting families to resources reducing exposure risks. |
| Brooks (2021) | - Data informed placement of climate warning labels, guiding operator actions while authorities monitored implementation and evaluated message impact for advocacy |
| Bonell (2022) | - Data was used to highlight alternative cooking fuels and informing student‑led presentations and exhibitions, enabling youth to voice experiences, influence decisions, and engage with educators during an NGO‑supported climate solutions festival. |
| Symanski 2023 (2023) | - Data was used to guide a public health action plan, informing emission‑reduction frameworks, communication protocols, multilingual education, and leadership training, while partners used research findings to engage industry, support residents, and advocate for reduced metal exposures in affected neighbourhoods |
| Castillo 2024 (2024) | - Data was used to inform targeted indoor‑smoke mitigation guidance, supporting advocacy for HVAC upgrades, air purifiers, sealed indoor spaces, and protective masking during wildfire events |
| Zhang 2018 (2018) | - Data was used to inform advocacy by revealing pollution sources, enabling think‑tank engagement with policymakers and industry through structured dialogues that ultimately drove air‑quality policy change. |
| Slavik 2024 (2024) | - Data was used to communicate about wildfire smoke and air quality. |
| Okeowo 2025 (2025) | - Data from the Clean Air Hospital Framework (CAHF) was used to guide assessment of hospital operations, shaping interviews, inspections, and supplier surveys, enabling collaboration with city partners and engaging staff and patients to advocate for cleaner practices, sustainability commitments, and improved air‑quality initiatives |
| Castner 2022 (2022) | - Data was used to strengthen community advocacy, supporting closure of a polluting factory and redirecting collective action toward mitigating traffic‑related emissions. |
| Yorifuji (2016) | - Data was used to support Tokyo’s diesel‑control ordinance, while researchers applied quasi‑experimental methods to generate evidence that strengthened advocacy for stringent standards |
| Lau 2024 (2024) | - Data was used for Citywide air‑monitoring, exposure, and cohort data generated by researchers and health agencies informed advocacy for cleaner fuels, underpinning adoption of low‑emission bus and taxi programs and policies phasing out high‑polluting heating oils to reduce urban air pollutants. |
| Banerjee (2016) | - Data was used to guide development, testing, and deployment of cleaner biomass technologies and supporting the National Biomass Cookstoves Initiative to advance certification, monitoring, and research. |
| Climate and Clean Air Coalition (2023) | - Data was used to Create Young Scientist Programme supporting Youth Initiatives and trained youth on advocacy and decision-making on climate change |
